# American Dental Association

**Published:** 1886-10

**Authors:** 


### AMERICAN DENTAL ASSOCIATION.

TWENTY-SIXTH ANNUAL MEETING, HELD AT NIAGARA FALLS, AUG. 3, 4, 5 AND 6, 1886.

REPORTED FOR THE INDEPENDENT PRACTITIONER BY "MRS. M. W. J."

#### WEDNESDAY MORNING SESSION.

The meeting was called to order at 9.30, the President in the chair. The minutes of the preceding session were read and approved.

The Committee on Credentials reported delegates present from the following societies:

Illinois State Society.
California Odontological Society.
University of California.
Chicago Dental Society.
Michigan State Dental Association.
Louisiana State Dental Society.
Mississippi Valley Dental Society.

Odontological Society of Pennsylvania.
Sixth District Dental Society of New York.
Chicago Dental Club.
Connecticut Valley Dental Association.
First District Dental Society of New York.
Iowa State Dental Society.
Brooklyn Dental Society.
Eighth District Dental Society of New York.
Minnesota Hospital College, Dental Department.
Georgia State Dental Society.
Pennsylvania State Dental Society.
Indiana State Dental Association.
State University of Iowa, Dental Department.
Northwestern Dental Association.
New Orleans Odontological Society.
Pittsburgh Dental Association.

Dr. Ottofy, of Chicago, was added to the Executive Committee, to fill a vacancy.

Dr. Bödecker then introduced to the Association Dr. Wilhelm Herbst, of Bremen.

The President acknowledged the introduction, which, he said, was hardly needed, as all were acquainted with Dr. Herbst, by reputation at least. He bade him heartily welcome as a representative of German dentistry.

On motion of Dr. Atkinson, Dr. Herbst was made a member of the American Dental Association, by acclamation.

Dr. Herbst expressed in German, Dr. Bödecker translating, his appreciation of the welcome tendered him, which, he said, he was too much moved to express in words.

Dr. Crouse introduced Dr. Bryant, of Switzerland, and several members of the profession from Canada, to all of whom the privileges of the floor were extended.

On motion of Dr. Watkins, of Montclair, N. J., the thanks of the Association were tendered Dr. Bödecker, to whom the profession owed the visit of Dr. Herbst to this country.

The President remarked, in putting the motion to vote, that any man coming from abroad who can teach American dentists one single new thing should be warmly welcomed, for we were always anxious to obtain information concerning our life-work.

The discussion of Section I, Prosthetic Dentistry, Chemistry and Metallurgy, was continued. The influence of red rubber was further discussed by Drs. John Allen, Kingsley, Land, and others, but nothing new was elicited.

The subject was passed, and the report of Section II was called for.

On motion of Dr. Brophy, unanimous consent was given, and Section IV allowed to report, that Dr. Herbst might be heard on the subject of Operative Dentistry.

The report of Dr. Darby, chairman of Section IV, embraced a general review of the progress made in Operative Dentistry in the last quarter of a century; in the varieties of foil now in use, and the improvement in amalgams; in the use of the mallet, the rubber dam and the dental engine; in the preservation of pulps, and in the restoration of contour. In new appliances, he offered Dr. T. W. Brophy's band matrix, Dr. Fernandez's diamond drill, Dr. Swasey's matrix and rubber dam holder, Dr. Watkins' instruments for finishing amalgam fillings, and his new tooth brush. No papers having been presented to the Section, they asked the consideration of these subjects.

*Dr. Melotte*—Inquired why the appliances of Dr. Knapp were not named?

*Dr. Darby*—Replied that they had not been brought before the Section.

*Dr. Kingsley*—Said that they were purely mechanical, and do not pertain to Operative Dentistry. Had they been presented to that Section he should have protested, as, though very artistic, they were purely mechanical.

*Dr. Melotte*—Considered it an open question, and would be glad to hear other expressions of opinion.

*Dr. Morgan*—Said that, while the preparation of diseased roots required therapeutical treatment, and therefore belonged to operative dentistry, the manufacture of gold crowns and bridges was entirely mechanical, the operations requiring a blending of both branches.

*Dr. Melotte*—Insisted that a clear line should be drawn. According to that view, in general surgery the treatment of the stump of an amputated leg was operative, while the adjustment of the wooden leg was prosthetic surgery. In the same way the treatment of roots was dental surgery, while making the crown or the bridge was mechanical work.

*Dr. McKellops*—Said the question might be discussed all day; it would be somewhat difficult to say how much, in ordinary fillings, belongs to surgery, and how much is mere mechanical work.

*Dr. Parmly Brown*—Said that at Minneapolis it had been decided that crown and bridge-work belonged to operative dentistry.

*Dr. McKellops*—Said that it required as much brain and genius to apply an artificial limb in the proper position as to operate upon the limb, and the same was true of bridge-work. Every part of this belonged to operative dentistry, as much as the filling of a tooth with gold. Only a skilled operator was competent to place such an appliance in the mouth.

Prof. Taft here rose to a point of order, the subject of nomenclature not being before the body.

The President decided the point of order well taken, and the discussion was ruled out.

On motion of Dr. Fillebrown, Dr. Herbst was invited to address the Association. His remarks were again translated by Dr. Bödecker.

He remarked that he would not repeat what he had said in New York and Philadelphia. It was well known that he had come to this country to exhibit his method of packing gold in the cavities of teeth. By this method, gold is brought into contact by rubbing against the walls, obtaining the best possible adaptation, much better than by malleting. For the success of this method much depends upon the cavity, which must have four side walls. If the tooth has not got them, they can be supplied by means demonstrated in his clinics. The patient would certainly recognize with gratification the difference between the hammer and this method. All that is done in first-class fillings with the mallet can be accomplished by this method of rotation, and there is a decided gain in time required for the introduction of the gold, in the weight of gold introduced, in adaptation to the walls of the cavity, and the consolidation of the several layers with each other. He said it was well known that the best operators in the profession, the world over, are found in America, and he had come to this country believing they would be honest and generous with him. In Europe he had shown his method to a great many of his *confrères*, but as yet they had done but very little with it. While admitting that many American operators were men that the world cannot reproduce, very able men, who can do with their own methods more than he had ever been able to do with his, he nevertheless believed that with his method much good might be done. It was very much easier, more rapidly acquired, and demanded less skill than the mallet method. He did not believe, and did not hope that the mallet would ever be abandoned. In fact, he endorsed the advice of Dr. Bödecker, that it was safer for the beginner to use the mallet in the last layers of the filling. What he might say would be of very little consequence in comparison with the demonstrations which he hoped to give. Talking about it would not help very much, and he could not show it to all. The particular point was to be very careful about the first layer of gold introduced into the cavity. He said that he felt he was not going back without having accomplished some good. Some had comprehended his method thoroughly, and he would trust to them to show others. He begged them, however, not to throw it entirely away without a trial, not only for themselves, but for their patients, who would derive great satisfaction from it. He said that, to him, many of the best American operators looked fatigued and worn out. By practicing his method they would save time and not have to work so hard, while they would accomplish more, and do it easier. In conclusion, he hoped, in another year, to meet them all again in Washington, at the International Medical Congress.

*Dr. Ottofy*—Asked Dr. Herbst to state in a few words the advantages of his methods over ours; if better, in what respect is it so? He asked permission to address the question to Dr. Herbst in his own tongue.

*Dr. Bödecker*—Replied for Dr. Herbst, that the first claim was for better adaptation of gold to the walls of the cavity than could possibly be obtained by any other method, as had been proved in the New York clinics to the satisfaction of everyone who had examined the work then done, and also by the work done by Dr. Herbst several years ago. It was by the perfect adaptation of gold to the walls of the cavity that the tooth is saved. Gold may be hammered against smooth glass walls, and the microscope will show that the adaptation is not perfect, but if a soft piece of gold is put in, with tweezers or hand plugger, and with a rotating instrument pressed down, or by a rotating burnisher in the dental engine, and it then be examined under the microscope, it will be found that there is absolute contact. Glass shows this better, as the contact can be seen in all portions. If such a filling is put in carmine or aniline solutions, there will be no discolorations between the gold and the glass, while in the best malleted filling thin solutions of aniline will penetrate.

Again, one-half the time is saved. In the proximal surfaces of molars and bicuspids, extending under the gum, which are generally difficult and tedious, as a rule, great advantage is gained, the most difficult operations becoming the simplest of all. In the grinding surface of molars it is better to use the mallet for the last layer, as access is easy, and a wearing surface is more readily made with the mallet by beginners. Patients who have experienced both methods will certainly ask you to fill by the Herbst method, rather than suffer from the mallet. I know it from my own experience, and all those who have had work done by both methods will bear me out.

*Dr. Atkinson*—Said the exposition of this method would not be complete without giving the Herbst obtunder.

*Dr. Bödecker*—Stated that when Dr. Herbst told him of it two years ago, he then felt as though care must be exercised, and he did not know whether he would dare to bring it out. He was sorry now that he had not given it sooner, for his experience had shown it to be perfectly safe.

A little chemically pure sulphuric acid is saturated with hydrochlorate of cocaine, and stirred with a glass rod until perfectly dissolved. This will give a test of the chemical purity of cocaine. If the solution remains uncolored, it is pure; if the sulphuric acid gets dark, it shows impurities (as carbonaceous matter) in the cocaine. To this solution is added sulphuric ether to super-saturation, still stirring with a glass rod, and it is best done in a long test tube. If shaken in a bottle, the expansion is liable to break the bottle, or force the cork out, the contents perhaps flying in the eyes. Any excess of ether will evaporate by standing. A little of the preparation is to be taken on cotton and applied to the sensitive dentine. The obtundent effect is more prompt and perfect than with anything else that has been offered for the profession. It will obtund only one layer of dentine, when another application must be made, but its action is very prompt. Measurement is not necessary to obtain the proper proportions of cocaine and sulphuric acid, as the acid will not take up more cocaine than is requisite. The cocaine relieves the momentary pain caused by the application of the sulphuric acid.

*Dr. Taft*—Said that it had been his privilege to spend three days with Dr. Herbst. In regard to the obtundent, when the formula was given him it struck him as rather peculiar. He had taken it home and made it up carefully, and found that one drachm of sulphuric acid would dissolve thirty grains of crystals of cocaine. The amount of sulphuric ether was not important, as any excess would evaporate. He had used it daily for three months, and in every instance it had proved successful, and he believed it would prove generally efficacious. Its action is very simple. The sulphuric acid dissolves a portion of dentine; acting as a caustic on living tissue, the cocaine obtunds sensation during its action. The sulphuric ether serves the same purpose, relieving the pain caused by the application of the sulphuric acid. If the cavity has been formed before the application, there is no objection to leaving the powder formed of the dissolved dentine.

Though the Herbst method may not be generally adopted for all cases, there is much in it that can be utilized. It cannot be comprehended from printed instructions; it must be seen to be understood. Many will adopt it, to a large extent, for many operations and in many kinds of cavities. It is especially advantageous in cavities without angles or depressions, or points for anchorage. Sometimes, when a large mass of gold is rolled up and packed in, it is difficult to press it down and have perfect adaptation at all points; while condensing at one point it will withdraw at another; or if by another method little pieces are to be held down, it is difficult to secure uniform pressure everywhere. In such cases Dr. Herbst takes a pellet of cotton and fills the mouth of the cavity, and, holding it down, goes all over the cotton with a rotary pressure. On removing the cotton the cavity will be found gold-lined throughout, the adaptation being apparently perfect—though we should be very careful in the use of that word. If we take a stone in which there is fine engraving, this method may be employed to bring out the finest lines of carving. Gold is laid on and rubbed down through the cotton, when the carving is perfectly reproduced in the gold; it is pressed into the mi[illegible] lines. If only for this perfect adaptation to the walls of the cavity, it is well worth trying.

*Dr. Marshall*—Said that, having been a patient of Dr. Herbst, his experience might be of interest. He was ready to say that, as a patient, he preferred the Herbst method to the mallet; the suffering was less than one-half as severe. The cavities filled were a molar crown cavity, and a compound distal and grinding surface of a bicuspid. The pressure was very heavy, as heavy as hand pressure in cylinder fillings; so much so that his jaws ached and he had to support his chin. The pressing and drawing motion gave a sharp click as it came against the walls of the cavity, and the feeling was as if the wall was broken by the blow. It was, however, only the sensation of the peculiar motion.

*Dr. Atkinson*—Said that Dr. Bödecker wrote him from Bremen that he had witnessed something new, which he would love. He had thought that he would never need anything but the mallet, but not until now had he really comprehended what the rotary method meant. He had witnessed sixteen clinics, four of which he had seen all through, one being in his own mouth, so that he knew both ends of the instrument. He was now ready to say that he had received a new revelation. There was never anything to equal it for surface fillings; he had tested it and knew it to be the densest he had ever seen. He had induced Dr. Herbst to try number 120 rolled gold, and it had worked beautifully in a skeleton, and in the jaw. Then he tried platinum and iridium-gold for wearing edges, and succeeded with that also. He recommended anyone, if he did not succeed, to go to some one who had been indoctrinated, and learn of him. He had feared the elimination of heat from rapid rotation, but had worked with a slow motion of the engine, giving a peculiar twist with the thumb and forefinger. Anyone who had learned hand-pressure could easily pack towards the wall of the cavity. The sensation of the agate point striking against the edge of the tooth was startling at first. His last words would be, should we never meet again, try it until you comprehend it, and you will never abondon it.

*Dr. Marshall*—Said (of friction and heat) that in the first cavity he had experienced no sensation of heat, but in the second, whether from moisture or what not, the gold lost its cohesion, and he thought it [illegible] have to come out. He took a heavy instrument and rotated it rapidly, producing sufficient heat to restore cohesion.

*Dr. Rehwinkle*—Said that from a sense of gratitude to Dr. Herbst, and of duty to the profession, he would tell what he knew of the Herbst method. Three years ago he first heard of it through the German journals. In going through their contents he found the report of a clinic by Herbst, at Frankfurt-on-the-Main, and exclaimed to himself: "Hello! here is a new method, by rotation; rubbing in the gold; it is certainly fascinating in theory." So he went home, and, guided by the description thus given, began to experiment, using tin foil to avoid the waste of gold. He pegged away, but not having any standard of comparison did not know whether he was on the right road or not. Learning that Dr. Herbst had shown his kindness and self-sacrifice by going from one office to another to exhibit his method, he wrote to him for specimens showing the steps of the process.

The response was prompt, and with his characteristic generosity, he sent, not only a full line of specimens, showing all the stages of the operation, but also a set of his instruments, matrices, etc.

The only question, and one which time alone can answer, is whether it will prove durable. He would not repeat what had been already said as to the method of operating, or the apparent perfection of the work, but could fully corroborate it all. If used only as a lining for cavities, preventing the discoloration of amalgam against thin borders, it will be a great acquisition. The cavity can be thoroughly lined with gold, and the body of the filling made with amalgam. In this case, the surface of the gold should be painted with a thin solution of copal varnish, to prevent it from being affected by the mercury in the amalgam. Dr. Herbst can make a gold filling by his method in one-fourth the time it would take any other man by any other method, It gives close, or perfect adaptation, using this word in its general sense. Coming from the same country, and from the neighborhood of Dr. Herbst, Dr. Rehwinkle offered that as his apology if he seemed too enthusiastic about the man and his method, and especially his great liberality in freely giving all he has for the benefit of the profession.

Dr. Bödecker, by request, gave again, in full detail, the manner of operating, from the preparation of the cavity to the surface finishing.

*Dr. C. N. Pierce*—Said he felt satisfied that the results would prove of great value. Even if not generally adopted, the younger members should take it up and study it faithfully, not being discouraged if their first efforts failed, remembering that although Herbst himself had been using it for seven years, he admitted that it was only within six months that he had become fully satisfied with the results in certain cases, especially in contour work. He advised beginners to experiment on cavities in bone, or a tooth brush handle.

*Dr. Watkins*—Spoke with great satisfaction of fillings which Dr. Bödecker had made for him, by this method, two years ago, which have been exposed to severe service in mastication. A large filling in the anterior proximal surface of the third molar was very satisfactory; this was finished with the bloodstone instrument.

*Dr. Bryant* of Switzerland—Said that the method had proved very acceptable in his country, its special features being its great adaptability. For matrices he used very thin stumps of steel, such as watchmakers use for supporting the pendulum of clocks. It is extremely thin, and can be cut with scissors like paper, and it will go where silk floss cannot pass.

*Dr. Morgan*—Was not so sanguine of the anticipated benefits to be derived from the introduction of such an innovation.

*Dr. Jas. Truman*—Foresaw great danger to pulp life in the indiscriminate use of a dangerous escharotic as an obtunder of sensitive dentine.

*Dr. Bogue*—At this point said he would like, from the depths of his own ignorance, to ask *what the Herbst method is?* He would be much obliged for a clear definition.

The President asked Dr. Bödecker to reply, and he did so, with additional explanation from Dr. Rehwinkle.

*Dr. Bogue*—Did not think that the principles underlying the method had yet been reached, except in so far as it was a revival of the principles laid aside twenty-five or thirty years ago, in the hands of a very dexterous operator. Not a single new principle had been evolved. He had seen one hour and ten minutes consumed on a gold filling which he himself could have made in twenty minutes, with soft gold. He was glad to hear Dr. Rehwinkle say that Dr. Herbst was a modest man, but with all his claims his principles were as old as the hills. It was the old story of the revolving cycles.

*Dr. Fillebrown*—Said that in the discussion, everything but Herbst *versus* the mallet was left out of consideration, as though there had never been any intermediate method used. But there were operators who made very creditable operations without the mallet and without the matrix, and who had never tried the Herbst system. They had found that great force was not essential; that it was not necessary to hammer all the life out of both the gold and the tooth; that light hand pressure would produce a hard surface that would wear for years, resisting both the force of mastication and the inroads of fluids.

*Dr. Taft*—Thought the reasoning of Dr. Truman on the action of the new obtunder was hardly borne out by the facts. Sulphuric acid has been used for years in surgery, and its action is well understood. An agent so violent as is portrayed by Dr. Truman would long ago have been found utterly impracticable. While it is true that it is an escharotic, it destroys only one layer, and then it stops. It is self-limiting, and not capable of doing the mischief portrayed by Dr. Truman. His only fear was that some might be deterred from using an agent capable of rendering great service.

*Dr. Rehwinkle*—Added that Veratria was also a valuable obtunder, but should be used with great caution, as it is a dangerous poison. One grain of Veratria is put in three drops of absolute alcohol, and sufficient tannin added to saturate; ten drops of glycerine is then added. It is to be applied after the dam is in place and the cavity dried.

*Dr. Bödecker*—Affirmed the perfect safety of Dr. Herbst's obtundent, having seen its excellent effects in teeth of very delicate structure, of children ten or twelve years of age, which have stood gold fillings perfectly for several years, with no bad results whatever, an exposed pulp even, having survived, and the edges of the filling were found absolutely perfect. They were teeth in which he would not have put gold under any circumstances, but Dr. Herbst considered that gold fillings, after the application of this remedy, were the best that could be used to preserve delicate teeth.

*Dr. Taft*—Said that the action of the sulphuric acid was to break up the calcareous surface of the cavity, forming a layer of sulphate of lime, an insoluble powder, the cocaine protecting the exposed organic portions of the tooth. Its action was not capable of being carried any further. The acid is satisfied with what it has dissolved, and the powder formed is insoluble.

*Dr. Atkinson*—Said that chemists themselves were sometimes at loose ends about their combinations, but in dealing empirically, if uniformly successful, no harm was done. We should not be alarmed by old fogies who don't know the first letters of the alphabet of chemistry. Some agents are self-limiting, and we know exactly what they will do. Sulphuric acid is one of these. Of others, however, we do not know where the mischief will end. Chloride of zinc quiets a nerve by transforming the pulp into hypochloride of albumen—as clear as glass quite to the apex. He wished to speak in defence of the man who cannot speak English for himself, and he only wished he could speak German to tell him how much he loves and admires him, not as a personality, but for his principles. If we would only put ourselves in the line of reception, our minds would soon be convinced of what is true light and wisdom.

Adjourned.

